# CyberKnife radiosurgery for inoperable stage IA non-small cell lung cancer: 18F-fluorodeoxyglucose positron emission tomography/computed tomography serial tumor response assessment

**DOI:** 10.1186/1756-8722-3-6

**Published:** 2010-02-04

**Authors:** Saloomeh Vahdat, Eric K Oermann, Sean P Collins, Xia Yu, Malak Abedalthagafi, Pedro DeBrito, Simeng Suy, Shadi Yousefi, Constanza J Gutierrez, Thomas Chang, Filip Banovac, Eric D Anderson, Giuseppe Esposito, Brian T Collins

**Affiliations:** 1 Department of Radiation Medicine, Georgetown University Hospital, Washington, DC, USA; 2 Department of Pathology, Georgetown University Hospital, Washington, DC, USA; 3 Division of Pulmonary, Critical Care and Sleep Medicine, Georgetown University Hospital, Washington, DC, USA; 4 Department of Nuclear Medicine, Georgetown University Hospital, Washington, DC, USA; 5 Department of Radiology, Georgetown University Hospital, Washington, DC, USA; 6 Division of Vascular & Interventional Radiology, Georgetown University Hospital, Washington, DC, USA

## Abstract

**Objective:**

To report serial 18F-fluorodeoxyglucose (^18^F-FDG) positron emission tomography (PET)/computed tomography (CT) tumor response following CyberKnife radiosurgery for stage IA non-small cell lung cancer (NSCLC).

**Methods:**

Patients with biopsy-proven inoperable stage IA NSCLC were enrolled into this IRB-approved study. Targeting was based on 3-5 gold fiducial markers implanted in or near tumors. Gross tumor volumes (GTVs) were contoured using lung windows; margins were expanded by 5 mm to establish the planning treatment volumes (PTVs). Doses ranged from 42-60 Gy in 3 equal fractions. ^18^F-FDG PET/CT was performed prior to and at 3-6-month, 9-15 months and 18-24 months following treatment. The tumor maximum standardized uptake value (SUV_max_) was recorded for each time point.

**Results:**

Twenty patients with an average maximum tumor diameter of 2.2 cm were treated over a 3-year period. A mean dose of 51 Gy was delivered to the PTV in 3 to 11 days (mean, 7 days). The 30-Gy isodose contour extended an average of 2 cm from the GTV. At a median follow-up of 43 months, the 2-year Kaplan-Meier overall survival estimate was 90% and the local control estimate was 95%. Mean tumor SUV_max _before treatment was 6.2 (range, 2.0 to 10.7). During early follow-up the mean tumor SUV_max _remained at 2.3 (range, 1.0 to 5.7), despite transient elevations in individual tumor SUV_max _levels attributed to peritumoral radiation-induced pneumonitis visible on CT imaging. At 18-24 months the mean tumor SUV_max _for controlled tumors was 2.0, with

a narrow range of values (range, 1.5 to 2.8). A single local failure was confirmed at 24 months in a patient with an elevated tumor SUV_max _of 8.4.

**Conclusion:**

Local control and survival following CyberKnife radiosurgery for stage IA NSCLC is exceptional. Early transient increases in tumor SUV_max _are likely related to radiation-induced pneumonitis. Tumor SUV_max_values return to background levels at 18-24 months, enhancing ^18^F-FDG PET/CT detection of local failure. The value of ^18^F-FDG PET/CT imaging for surveillance following lung SBRT deserves further study.

## Introduction

Stereotactic body radiation therapy (SBRT) is an accepted treatment for inoperable stage I NSCLC [1-12]. Several techniques have been employed to treat these potentially mobile tumors with relatively tight margins (5-10 mm). This enhanced accuracy has facilitated the safe, swift delivery of extremely high radiation doses. As anticipated, such treatment has improved local control and overall survival rates relative to historical controls. However, high peritumoral lung doses have resulted in focal radiation-induced pneumonitis and fibrosis, hampering the assessment of the tumor response using CT and ^18^F-FDG PET imaging [13-17]. To date, a reliable noninvasive means of detecting early local failure following SBRT remains to be established.

In mid-2004 we opened a novel thoracic stereotactic radiosurgery treatment protocol for patients with inoperable small peripheral lung tumors [18,19]. The enhanced accuracy and flexibility of the CyberKnife [20,21] facilitated the safe delivery of dose distributions designed to eradicate both gross tumor and known microscopic disease radiating from it [22]. Twenty patients with peripheral clinical stage IA NSCLC were treated in 36 months. As anticipated, given the small tumor size and peripheral location, both overall survival and local control were excellent. However, such treatment did result in focal peritumoral pneumonitis and fibrosis, which interfered with CT tumor response assessment [18,19]. We followed this patient cohort for a minimum of 18 months and evaluated the change in tumor maximum standardized uptake value (SUV_max_) following radiosurgery at 3-6 months, 9-15 months and 18-24 months.

## Methods and materials

### Eligibility

This study was approved by the hospital institutional review board. Patients consecutively treated on a single institution prospective protocol with inoperable

biopsy-proven peripheral clinical stage IA NSCLC were evaluated. Inoperability was defined as a post-operative predicted forced expiratory volume in one second (FEV1) of less than 40%, post-operative predicted carbon monoxide diffusing capacity (DLCO) of less than 40%, VO_2 _max less than 10 ml/kg/min, age greater than 75, or severe comorbid medical conditions. Pure bronchioloalveolar carcinomas were excluded.

### Treatment Planning and Delivery

Patients were treated according to the Georgetown University Hospital small peripheral pulmonary nodule protocol as previously described [18,19]. Briefly, fine-cut (1-mm) treatment planning CTs were obtained 7-10 days after CT-guided percutaneous biopsy and fiducial placement. Gross tumor volumes (GTV) were contoured utilizing lung windows. The GTV margin was expanded 5 mm to establish the planning treatment volume (PTV). A treatment plan was generated using the CyberKnife non-isocentric, inverse-planning algorithm with tissue density heterogeneity corrections for lung. The radiation dose, ranging from 42-60 Gy in 3 fractions, was prescribed to an isodose line that covered at least 95% of the PTV and resulted in the 30-Gy isodose contour extending a minimum of 1 cm from the GTV.

Subsequently, patients were brought to the CyberKnife suite and laid supine on the treatment table with their arms at their side. Three red light-emitting diodes (LEDs) were placed on the patient's anterior torso directed toward the camera array. Fiducials were located using the orthogonal x-ray imagers. A correlation model was created between the LEDs tracked continuously by the camera array and the fiducial positions imaged periodically by the x-ray targeting system. During treatment delivery the tumor position was tracked using the live camera array signal and correlation model; the linear accelerator was moved by the robotic arm to maintain precise alignment with the tumor throughout the respiratory cycle. Fiducials were imaged prior to the delivery of every third beam to verify targeting accuracy and to update the correlation model.

### Follow-up Studies

Patients were followed per institutional protocol [18,19]. ^18^F-FDG PET/CT imaging was performed prior to and at 3-6, 9-15 and 18-24 months following radiosurgery. CT was used for attenuation correction of the PET emission image data. Quantitative values of tumor metabolic activity were collected by the first author and expressed as tumor SUV max, defined as the maximum standardized uptake value within the tumor. Values were obtained using three-dimensional regions of interest placed on the lung lesions, which were anatomically defined by combined review of the PET and CT images. Local tumor recurrence was defined as unequivocal progression on serial ^18^F-FDG PET/CT imaging. Biopsy was required to confirm recurrence.

### Statistical Analysis

Data was analyzed and graphs were prepared with the SPSS 16.02 statistical package. The follow-up duration was defined as the time from the date of completion of treatment to the last date of follow-up or the date of death. Actuarial survival and local control were calculated from the conclusion of treatment using the Kaplan-Meier method.

## Results

### Patient Characteristics

Twenty consecutive predominately older female (4 men and 16 women) former heavy smokers with biopsy-proven clinical stage IA NSCLC (adenocarcinoma 8, NSCLC not otherwise specified 7 and squamous cell carcinoma 5) were treated over a 3-year period extending from January 2005 to January 2008 (Table 1). Surviving patients were followed for a minimum of 18 months without exception.

**Table 1:** Patient Characteristics

	Mean (Range)
Age (years)	75 (64-86)

Weight (lbs)	153 (116-225)

FEV1 (L)	1.12 (0.53-2.48)

% predicted FEV1	59 (21-111)

### Treatment Characteristics

The mean maximum tumor diameter was 2.2 cm (range, 1.4 - 3.0 cm) and the mean gross tumor volume (GTV) was 10 cc (range, 4 - 24 cc). Treatment plans were composed of hundreds of beams shaped using a single circular collimator (20 to 30 mm in diameter). The mean dose delivered to the prescription isodose line in three equal fractions over an average of seven days was 51 Gy (Table 2). The 30-Gy isodose contour, biologically equivalent to 50 Gy in 2-Gy fractions, extended an average of 2 cm from the GTV (range, 1.08 - 2.74 cm).

**Table 2:** Treatment Characteristics

	Mean (Range)
Prescribed Dose (Gy)	51 (42 - 60)

Prescription Isodose Line (%)	80 (75 - 85)

Prescribed Biologic Effective Tumor Dose (BED Gy_10_)	141 (100-180)

Prescribed Biologic Effective Lung Dose (BED Gy_3_)	380 (270-460)

Treatment course (days)	7 (3-11)

### Disease Spread and Survival

**Figure 1:**
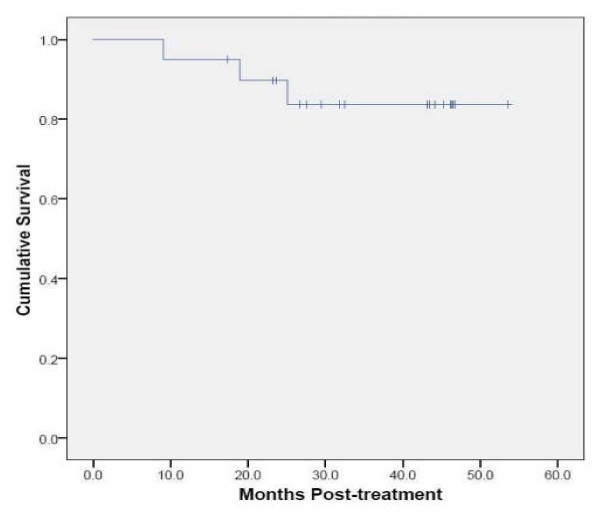
**Kaplan-Meier plot of overall survival**.

No regional lymph node failures have been observed. Three patients are alive with distant lung metastases. Deaths have been attributed to progressive lung dysfunction at 9, 18 and 25 months, respectively. Therefore, with a median follow-up of 43 months, the 2-year Kaplan-Meier estimated overall survival is 90% (Figure 1).

### Serial change in SUV_max _and Local Control

**Figure 2:**
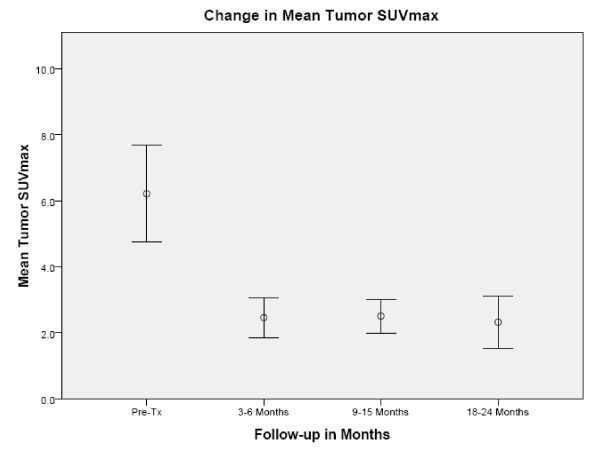
**Change in mean tumor SUV_max_**.

**Figure 3:**
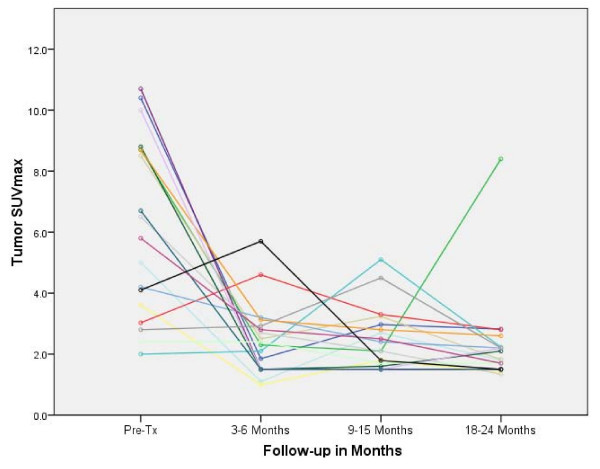
**Individual patient changes in tumor SUV_max_**.

**Figure 4:**
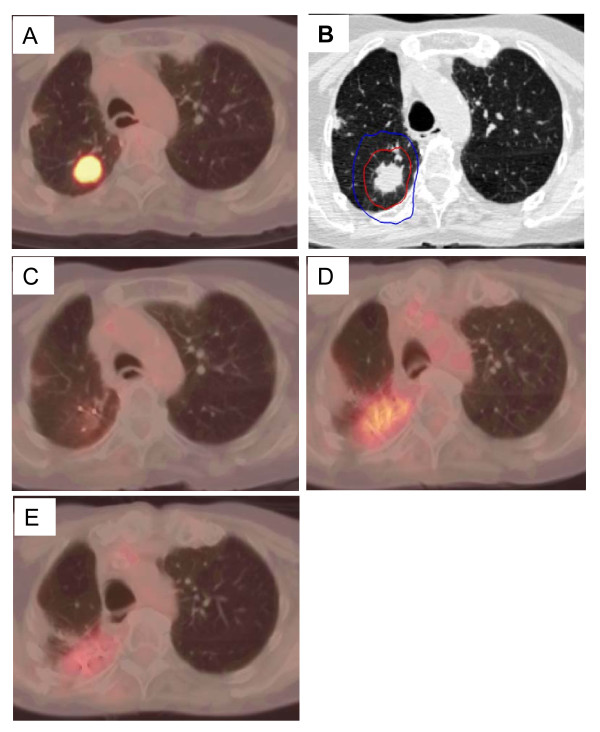
**Right upper lobe clinical stage IA NSCLC treatment planning PET/CT with a tumor SUV_max _of 10.5 (A), planned radiation dose distribution (B: the planning treatment volume receiving 45 Gy shown in red and the 30 Gy isodose line in blue), and PET/CT at 6, 12, and 18 months post-treatment (C, D and E) show an initial decrease in tumor SUV_max _to 1.5 followed by a transient radiation induced increase (tumor SUV_max _= 4.0) which resolves by 18 months (tumor SUV_max _= 2.5)**.

**Figure 5:**
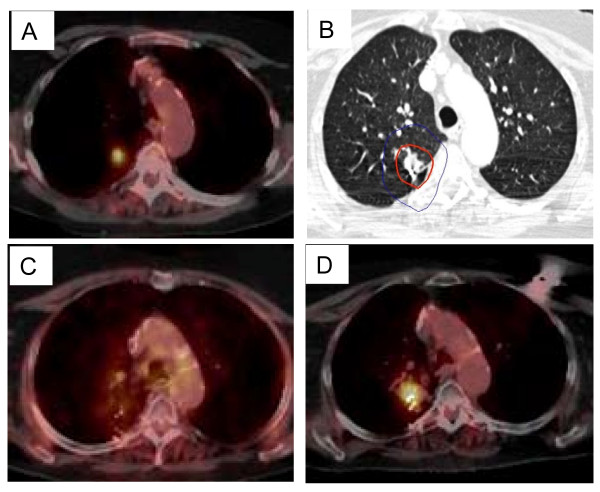
**Right upper lobe clinical stage IA NSCLC treatment planning PET/CT with a tumor SUV_max _of 8.7 (A), planned radiation dose distribution (B: the planning treatment volume receiving 45 Gy in red and the 30 Gy isodose line in blue), and PET/CT at 12, and 24 months post-treatment (C and D) show an initial decrease in SUV_max _to 2.3 followed by local recurrence (SUV_max _= 8.4)**.

**Figure 6:**
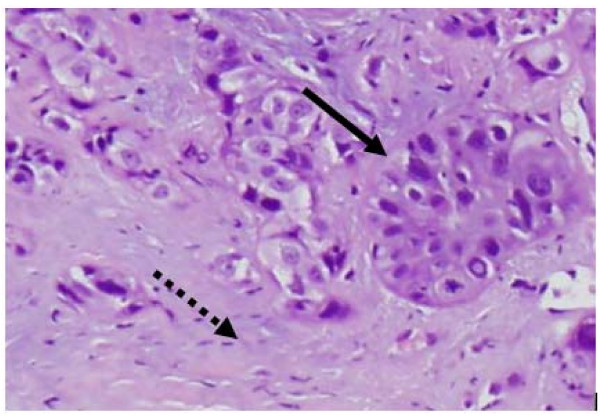
**Recurrent tumor (bold arrow) infiltrating radiation-induced lung fibrosis (dashed arrow)**.

**Figure 7:**
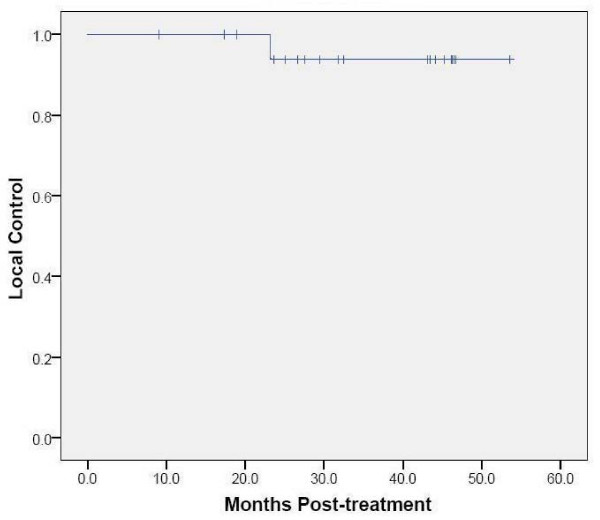
**Kaplan-Meier plot of local control**.

The mean tumor SUV_max _before treatment was 6.2 (range, 2.0 to 10.7). Fifty-seven of sixty planned post treatment ^18^F-FDG PET/CT studies were completed and reviewed. At 3-6 months there was a decrease in the mean tumor SUV_max _to 2.3 (range, 1.0 to 5.7), where it settled for the remainder of the analysis despite fluctuations in some tumors attributed to peritumoral radiation-induced pneumonitis visible on CT imaging (Figure 2). Transient tumor SUV_max _elevations, occurring as early as 3 months following treatment and as high as 5.7, uniformly peaked prior to the 18-24 month ^18^F-FDG PET/CT imaging (Figure 3, 4). A single local failure within the PTV was pathologically confirmed at 24 months in a patient with what appeared to be typical benign peritumoral lung fibrosis per CT imaging but an elevated tumor SUV_max _of 8.4 (Figure 3, 5). Microscopic evaluation revealed recurrent tumor infiltrating radiation-induced lung fibrosis (Figure 6) and salvage radiofrequency ablation (RFA) was completed. Therefore, with a median follow-up of 43 months, 2-year Kaplan-Meier estimated local control with CyberKnife radiosurgery is 95% (Figure 7.)

## Discussion

Prior to initiating the CyberKnife radiosurgery protocol for inoperable stage IA NSCLC patients, published reports had documented deficiencies with CT tumor response assessment following SBRT [13,14]. SBRT delivered to small peripheral lung tumors with adequate margin damages peritumoral lung tissue and often causes acute radiation pneumonitis [23]. Repair of the lung injury typically results in asymptomatic focal lung parenchyma fibrosis in the region that corresponds with the high-dose radiation volume [13,14]. As expected, CT imaging evidence of focal radiation-induced pneomonitis and fibrosis was consistently observed within the target volumes of our patients during follow-up as well [18,19]. ^18^F-FDG PET/CT is the standard imaging tool for NSCLC at Georgetown University Hospital. It is both more sensitive and specific than conventional imaging for the detection of primary lung tumors, involved regional lymph nodes and distant metastases [24,25]. Primary lung tumors with a SUV_max _greater than 2.5 are considered malignant until proven otherwise. However, preliminary evidence suggested that like CT imaging, ^18^F-FDG PET imaging is limited in assessing local tumor control following SBRT due to early elevations in tumor SUV_max_, which are thought to be related to acute radiation-induced pneomonitis [15]. Therefore, prior to starting protocol therapy, the decision was made to routinely observe inoperable patients with early transient elevations in tumor SUV_max _following radiosurgery.

The mean tumor SUV_max _before treatment was 6.2 (range, 2.0 to 10.7), consistent with the small size and mobility of treated tumors. Study compliance was excellent with 95% of planned surveillance ^18^F-FDG PET/CT scans being completed. Although we observed an initial sharp decline in mean tumor SUV_max _to 2.3 followed by stable mean tumor SUV_max _levels during the remainder of the 18-24 month follow-up, individual tumors frequently showed transient moderate SUV_max _elevations (Figure 3) that were always closely correlated with delivered radiation dose distributions and CT evidence of radiation pneumonitis (Figure 4). ^18^F-FDG PET/CT imaging at 24 months detected our single local recurrence (Figure 5). High tumor SUV_max _alone prompted immediate biopsy, which confirmed recurrent tumor infiltrating radiation-induced lung fibrosis (Figure 6). Following the 18-24 month evaluation no tumor SUV_max _elevations or local failures were identified in this patient cohort with a median follow-up of 43 months and excellent survival despite routine ^18^F-FDG PET/CT imaging (Figure 7).

In contrast to previous investigations, this study is reported with both serial imaging and adequate follow-up [15-17]. Nonetheless, a critical issue concerning its validity, and the validity of all other currently available studies like it, merits serious consideration. Routine biopsy was not justified in this study given the uncertain clinical significance of early transient elevations in tumor SUV_max _following radiosurgery and the risk associated with biopsy in this inoperable patient population with limited salvage treatment options. Therefore, confirmation of radiographic impressions was limited to a single biopsy in one patient following an increase in tumor SUV_max_; biopsies were not taken to confirm the absence of the disease in cases in which tumor SUV_max _remained low. Therefore, it is likely that the true local control rate in this study is less than our reported 95% rate. Furthermore, this difference in local control rates could be considerable in patients with low pre-treatment tumor SUV_max _values.

Future research, enrolling operable patients with effective salvage surgery options, will mandate routine biopsy. These studies will ultimately determine the clinical utility of surveillance ^18^F-FDG PET/CT imaging following lung radiosurgery. In the interim, it remains our institutional practice to complete routine serial surveillance ^18^F-FDG PET/CT imaging following radiosurgery for stage IA NSCLC to detect local, regional and metastatic disease.

## Conclusions

Local control and survival following CyberKnife radiosurgery for stage IA NSCLC is exceptional [18,19]. However, high planned peritumoral lung doses result in acute radiation induced pneumonitis, which hinders early ^18^F-FDG PET/CT tumor response assessment [12-16]. It appears that tumor SUV_max _values return to background levels at 18-24 months, enhancing ^18^F-FDG PET/CT detection of local failure. The value of ^18^F-FDG PET/CT imaging for surveillance following lung SBRT deserves further study.

## Abbreviations

BED Gy_3_: biologic effective lung dose; BED Gy_10_: biologic effective tumor dose; CT: computed tomography; ^18^F-FDG: 18F-fluorodeoxyglucose; GTV: gross tumor volume; Gy: Gray; NSCLC: non-small cell lung cancer; PET: positron emission tomography; PTV: planning treatment volume; SBRT: stereotactic body radiation therapy; SUV_max_: maximum standardized uptake value.

## Competing interests

BC is an Accuray clinical consultant. EA is paid by Accuray to give lectures.

## Authors' contributions

SV participated in data collection, data analysis and manuscript revision. EO participated in data collection, data analysis and manuscript revision. SC prepared the manuscript for submission, participated in data collection, data analysis and manuscript revision. XY participated in treatment planning, data collection and data analysis. MA assisted pathologic analysis and created a Figure. PD performed pathologic analysis. SS created tables and figures and participated in data analysis and manuscript revision. SY participated in data analysis and manuscript revision. CG participated in data collection, data analysis and manuscript revision. TC participated in treatment planning, data collection, data analysis and manuscript revision. FB participated in treatment planning, data collection, data analysis and manuscript revision. EA participated in treatment planning, data collection, data analysis and manuscript revision. GE participated in data collection, data analysis and manuscript revision. BC drafted the manuscript, participated in treatment planning, data collection and data analysis. All authors have read and approved the final manuscript.
